# Determinants of COVID-19 vaccine acceptance in healthcare workers in Iran: National Survey

**DOI:** 10.1186/s12879-022-07675-x

**Published:** 2022-08-22

**Authors:** Koorosh Kamali, Zarrintaj Hoseinzade, Khadijeh Hajimiri, Soodabeh Hoveidamanesh, Seyed Mohsen Zahraei, Mohammad Mehdi Gouya, Sousan Mahmoudi Bavandpouri, Tahereh Mohamadi, Soraya Mohamadi, Zohre Bigdeli, Azam Maleki, Mahboubeh Shirzad, Zahra Heidari, Mahya Farsadegi, Alireza Shoghli

**Affiliations:** 1 School of Public Health, Social Determinants of Health Research Center, Zanjan University of Medical Sciences, Zanjan, Iran; 2 Social Medicine, School of Medicine, Social Determinants of Health Research Center, Zanjan University of Medical Sciences, Zanjan, Iran; 3 Health Education and Promotion, School of Public Health, Zanjan University of Medical Sciences, Zanjan, Iran; 4 Community Medicine, Burn Research Center, Iran University of Medical Sciences, Tehran, Iran; 5 Center for Communicable Diseases Control, Ministry of Health and Medical Education, Tehran, Iran; 6 Infectious Disease, School of Medicine, Iran University of Medical Sciences, Teheran, Iran; 7 Business Administration, Health Insurance Organization, Zanjan, Iran; 8 Biostatistics, Zanjan University of Medical Sciences, Zanjan, Iran; 9 Biostatistics, Social Determinant of Health Research Center, Zanjan University of Medical Sciences, Zanjan, Iran; 10 Reproductive Health, Social Determinants of Health Research Center, Zanjan University of Medical Sciences, Zanjan, Iran; 11 Social Determinant of Health Research Center, Zanjan University of Medical Sciences, Zanjan, Iran; 12 School of Dentistry, Zanjan University of Medical Sciences, Zanjan, Iran

**Keywords:** COVID-19, Vaccine hesitancy, Vaccine acceptance, Healthcare workers, Iran

## Abstract

**Background and aim:**

It seems that acceptance of COVID-19 vaccination is the most effective way to tackle the COVID-19 pandemic now. Health care workers (HCWs) are one of the most important groups who are at risk for COVID-19 infection. This study aimed to assess the COVID‐19 vaccine acceptance among HCWs in Iran and its determinants.

**Methods:**

A cross‐sectional survey was carried out among 3600 HCWs in Iran. Data were collected through a self-administered questionnaire by a trained team from February to March 2021. Multi-stage cluster sampling method was used for selecting respondents of the study. Multivariate logistic regression analysis was used to determine the key factors of COVID-19 vaccine acceptance among participants. P-value < 0.05 was considered statistically significant.

**Results:**

Out of the 3536 respondents, 2191 (62.1%) intended to uptake the COVID-19 vaccine. Only about 10 percent of respondents said they did not trust any vaccine (domestic or foreign). Willing to accept a COVID-19 vaccine was relatively high among males, doctors, and those who had a history of hospitalization due to COVID-19 infection. The multivariate regression analysis showed respondents who were 40–50 years (aOR: 1.56; 95% CI: 1.47–1.66), had a history of COVID-19 infection (aOR: 0.85; 95% CI: 0.83–0.88), and hospitalized due to COVID-19 infection (aOR: 2.18; 95% CI: 1.97–2.39), were significantly associated with vaccine acceptance (p < 0.05).

**Conclusion:**

Our study showed moderate acceptance of COVID-19 vaccination in the HCWs in the Islamic Republic of Iran. The most important factor in the acceptance of the COVID-19 vaccine by the health staff is having a history of hospitalization. Further training and justification of health personnel is needed to increase the acceptance of COVID 19 vaccine.

**Supplementary Information:**

The online version contains supplementary material available at 10.1186/s12879-022-07675-x.

## Introduction

Beyond dispute, our life has been twinned with a newly emerged respiratory disease (COVID-19) since 2019. The pandemic has posed a significant threat to the human lives and well-being of billions of people worldwide which caused a plethora of death around the world [1]. Various strategies have been implemented to tackle the COVID-19 pandemic, such as physical distancing measures and movement restrictions (lockdowns) in various countries, but the pandemic is still ongoing despite such efforts [2]. In order to combat this unknown disease, World Health Organization (WHO) declared that the most effective way to hold this problem is vaccination [3]. In other words, vaccines are critical to prevent and control infectious diseseses outbreaks, which can save millions of lives [4]. However, approved vaccine that were demonstrated to be safe is a critical point of vaccine acceptance [5]. Whereas vaccinating most people around the world with a COVID-19 vaccine is a controversial issue among people [6]. Vaccine acceptance among the general public plays a fundamental role in successfully controlling the pandemic [7]. There could be found many reasons why some people cannot trust the vaccine and still refuse to take it despite the availability of vaccination services [7]. One of those associated with the attitude towards acceptance of vaccination includes complacency, convenience, and confidence [7]. Therefore, vaccination may be deemed unnecessary. Health care providers are the most vulnerable group at risk of COVID-19 because they are in touch with COVID-19 patients [8]. Consequently, achieving a high vaccination coverage rate in this important target group is mandatory [8], and there is a pressing need to consider. Also, this group has one of the strongest influences in vaccination decisions, which can be vital in helping the acceptance of vaccines [9]. Besides, health care providers are a vital source of information for acceptance of vaccines, and their communication can improve adherence to vaccination recommendations among the public [10]. According to studies that have been conducted during this pandemic, delay acceptance in vaccination is a common phenomenon among the public. Perceived risks vs. benefits, certain religious beliefs and lack of knowledge and awareness are extracted as a common reason for it [11–13].

A study has shown that acceptance of a COVID-19 vaccine in the USA is 67% among general population. However, before introducing a COVID-19 vaccine for massive, lawmakers and the Ministry of health should plan a program to educate people on the effective COVID-19 vaccine acceptance [10].

One of the most effective measures against COVID-19 is immunization programs with high rates of acceptance and coverage. To accomplish this, it is crucially significant to understand the health care provider’s perceptions about acceptance of a COVID-19 vaccine. According to the results of a systematic review study, the vaccine acceptance rate among physicians and nurses varied between 27.7% and 78.1% [7]. Since the acceptance of the vaccine is a complex behavior; and rate of acceptance in different communities are affected by context, culture, time, place, perceived behavior, geography, and sociodemographic factors [14]. Therefore, this study aimed to identify the determinants of COVID-19 Vaccine Acceptance in Health Care Workers in Iran.

## Subjects and methods

### Design and sample

As part of a large scale research project, this national cross-sectional survey was conducted to assess the COVID-19 Vaccine acceptance in HCWs in Iran from February to March 2020. To ensure a sufficient level of accuracy of the survey findings, the multi-stage cluster sampling method was selected. To calculate the sample size, a proportion of participants willing to receive a COVID-19 vaccine set at 50%, confidence interval 95%, and design effect 1.7 was considered. The final estimated sample size was 3600.

In Iran, medical universities are also responsible for providing health services. From the list of the medical sciences universities of Iran, nine universities were selected randomly. Then, two general hospitals from each university, one of which was Corona center, and ten primary healthcare centers (rural and urban) were randomly selected. Respondents were selected by random sampling method according to healthcare workers statistics for gender and occupation from each university of medical sciences (n = 400). The flow chart of recruiting HCWs in Iranian Medical sciences Universities is presented in Additional file 1. Participants, those who worked in primary health care centers and hospitals, with at least three months of experience, were included.

### Procedures

Data were collected through a self-administered questionnaire by a trained team in a private office setting. Before data gathering, the purpose of the study was explained in detail for all participants by the research team. The anonymous questionnaires with no identifiable information were delivered to the participants, and they were assured that their participation was entirely voluntary. Informed consent was obtained from each participant.

### Data collection instruments

We developed a questionnaire based on the literature review. The draft was in Persian format and consisting of four parts. Qualitative face validity was assessed by 10 health workers. They assessed the content of the items, their comprehensibility, and suitability and any necessary modifications were made [15]. Content validity of the questionnaire was assessed by 10 experts working at the Zanjan University of Medical Sciences via calculating Content Validity Ratio (CVR) and Content Validity Index (CVI) for the items [16]. However, this scale had been piloted by a sample of 50 participants. The results of the pilot study were not included in the survey. The first part of the questionnaire consisted of individuals’ socio-demographic and general characteristics of participants such as age, gender, marital status, level of education, profession, workplace, work experience (years), location of health care service, history of COVID-19 infection, and history of hospitalization due to COVID-19 infection.

In the second part, participants’ likelihood to receive a COVID-19 vaccine was assessed by one expression “If COVID-19 vaccine is available, I will take it". Their agreement was rated on a 5-point Likert scale, with strongly agree = 1 to strongly disagree = 5. Based on the answers, participants were divided into three groups. Thus, if participants answered “strongly agree “and “agree “we consider them in ‘Intended to uptake COVID-19 vaccine’ group; If participants answered “disagree” and “strongly disagree” we consider them in ‘COVID-19 vaccine refusal’ group; and if participants answered” uncertain” we considered them ‘COVID-19 vaccine hesitancy’ group.

In the third part, participants who were intended to uptake COVID-19 vaccine or hesitate for vaccine uptake were asked to determine which type of vaccine they prefer to uptake: Vaccines which produced inside Iran (domestic), Vaccines which produced outside of Iran by other countries (foreign), and both of them. Also, they were asked whether they trusted the domestic or foreign COVID-19 vaccine. The types of answers to this question included: 1: Yes, if approved by the Ministry of Health of Iran; 2: Yes, if only scientific documentation is available and the Ministry of Health of Iran approves it; 3: Yes if approved by reputable international authorities; 4: Yes if only approved by reputable international authorities and the Ministry of Health of Iran; 5: No, I do not trust the COVID-19 vaccine.

In the final part, to assess the respondents’ sources of information on COVID-19, they were asked which sources of information they referred to. They could choose from provided list as many information sources as they wished. They also were asked to indicate who (individuals or organizations), as a reference, influence their decision to receive the COVID-19 vaccine. The respondents were able to indicate more than one answer regarding these questions too.

### Data quality control

After training of the data collectors, the data were collected under close supervision. Collected Data were entered into an excel datasheet. Before analysis, to ensure its authenticity, the data were double-checked by an independent team. In this stage, a number of 64 incomplete questionnaires were excluded from the analysis. Finally, the analysis was performed on the data of 3536 participants.

### Data processing and statistical analysis

Analysis of the data was done after checking for accuracy and completeness. At the next step, data exported to IBM^©^ SPSS^©^ Statistics version 22 (IBM^©^ Corp., Armonk, NY, USA). Then, the data were analyzed using appropriate descriptive statistics and were presented by frequency, percentage, and mean. Moreover, a Chi-square test was performed to examine the intention to uptake the COVID-19 vaccine with participants’ socio-demographic characteristics. Also, Multivariate Logistic Regression Model was performed to tabulate odds ratios (OR) and their 95% confidence intervals (95% CI). The P-value < 0.05 was considered statistically significant.

### Ethical consideration

Ethical approval was granted for the study protocol and procedures by the institutional Research Ethics Committee (IR.ZUMS.REC.1399.455), Zanjan University of Medical Sciences of Iran. In addition, informed consent was taken before participation in the study from all participants and all methods were carried out in accordance with relevant guidelines and regulations.

## Results

A total of 3600 study participants were enrolled in this study. Nevertheless, the analysis was performed on the data from 3525 respondents. Table 1 shows the socio-demographic characteristics of participants. The mean (SD) age and work experiences of respondents was 37.09 (8.54) and 11.9 (8.35) years, respectively. Most of the participants, 1336 (37.7%), were aged between 30 to 40 years; and the majority (54.9%) of them were female. 55. 1% of participants hold a bachelor's degree, 75.8% were married, and 35.5% reported a history of COVID-19 infection, whereas 3.7% hospitalized due to COVID-19 (Table 1).

**Table 1:** Socio-demographic characteristics of HCWs, Iran (N = 3536)

Variables	Frequency	Weighted percentage^a^
Sex		
Male	1582	45.1
Female	1953	54.9
Marital status		
Married	2709	75.8
Unmarried and widower	824	24.2
Age group, years		
< 30	980	27.6
30–40	1336	37.7
40–50	977	27.4
> 50	241	7.2
Education level		
High school, diploma	715	20
Bachelors degree	1956	55.1
Masters degree	306	8.8
MD	555	15.9
Profession		
Doctor	480	13.5
Nurse	838	23.4
Dentist	123	3.4
Paramedical staff	308	8.3
Nurse	249	7.1
Administrative staff of hospital	312	8.6
Health care personnel	436	12.7
Behvarz^b^	789	23
History of COVID-19		
Yes	1270	35.5
No	2261	64.5
History of hospitalization due to COVID-19		
Yes	130	3.7
No	3403	96.3
Work experience, year		
> 10	2013	57.5
≤ 10	1492	42.5
Place of work		
Hospital	1811	50.8
Primary health care center (urban)	876	24.4
Primary health care center (rural)	847	24.8

^a^Data in the weighted sample were weighted to the proportions of health care workers at each university obtained from the Ministry of Health of Iran

^b^Iranian rural health workers (Behvarz)

**Fig. 1:**
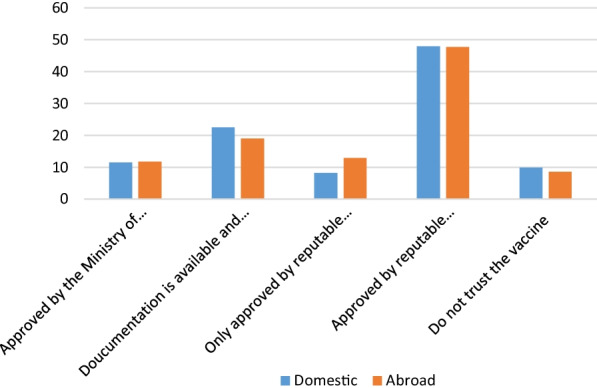
Condition of trust to uptake the COVID-19 vaccine based on the place of production among Iranian HCWs (N = 3536)

Participants who intended to uptake COVID-19 vaccine or hesitate for vaccine uptake were asked to determine which type of vaccine they prefer to Uptake; Our findings indicate that 40.4% of participants prefer foreign vaccines, which produced outside of Iran (other countries), 39.9% of them accept both domestic and foreign vaccines, and the remaining (28.7%) prefer merely domestic vaccine. Near 50% of the participants stated that they trust only a vaccine (either h domestic or foreign vaccine) that has been already approved by reputable international authorities and the Iranian Ministry of Health. Only 10% of participants said they did not trust any vaccine (neither domestic nor foreign) (Fig. 1).

The association between intention to uptake the COVID-19 vaccine and socio-demographic characteristics was examined by Chi-square test. Of the 3525 respondents, 2191 (62.1%) intended to uptake the COVID-19 vaccine, only 660 (18.7%) reported hesitancy regarding the COVID-19 vaccine, and 678 (19.2%) of them refused up taking the COVID-19 vaccine (Table 2). Of the 1582 participants who were male, 1063 (67.4%) of them showed a willingness to uptake the COVID-19 vaccine if it was available. Of the 2709 participants who were married, 1683 (62.3%) reported being willing to uptake the COVID-19 vaccination. While, 62.4% (n = 790) of respondents who had a history of COVID-19 infection stated that they were willing to receive a COVID-19 vaccine, and 20.2% (n = 256) stated that they would refuse the vaccination. (Table 2). The results of the study showed that among the socio-demographic characteristics of the participants, Sex, level of education and occupation had a significant effect on their intention to receive COVID-19 (P < 0.001). Also, the history of hospitalization due to COVID-19 had a significant effect on their vaccination intention (P < 0.007).

**Table 2:** Bivariate associations between socio-demographic characteristics and intent to uptake COVID-19 among Iranian HCWs (N = 3536)

Variables	“If COVID-19 Vaccine is Available, I Will Take It”			
	Intention to uptake COVID-19 vaccine (n = 2191, 62.1%)	COVID-19 vaccine refusal (n = 678, 19.2%)	COVID-19 vaccine hesitancy (n = 660, 18.7%)	p-value
Sex				
Male	1063 (67.4)	242 (15.3)	272 (17.2)	< 0.001
Female	1128 (57.8)	436 (22.3)	387 (19.8)	
Marital status				
Married	1683 (62.3)	522 (19.3)	498 (18.4)	0.763
Unmarried and widower	506 (61.5)	156 (19)	161 (19.6)	
Age group, years				
< 30	574 (58.6)	193 (19.7)	212 (21.7)	
30–40	845 (63.3)	248 (18.6)	242 (18.1)	0.084
40–50	614 (63.1)	195 (20)	164 (16.9)	
> 50	158 (65.8)	42 (17.5)	40 (16.7)	
Education level				
High school diploma	453 (63.6)	132 (18.5)	127 (17.8)	< 0.001
Bachelors degree	1165 (59.7)	400 (20.5)	387 (19.8)	
Masters degree	89 (55.9)	78 (25.5)	57 (18.6)	
MD^a^	399 (71.9)	67 (12.1)	89 (16)	
Profession				
Doctor	343 (71.5)	64 (13.3)	73 (15.2)	
Nurse	490 (58.5)	188 (22.5)	159 (19)	
Dentist	87 (70.7)	16 (13)	20 (16.3)	
Paramedical staff	191 (62)	61 (19.8)	56 (18.2)	
Practical nurse	164 (65.9)	47 (18.9)	38 (15.3)	< 0.001
Administrative staff of Hospital	163 (52.6)	75 (24.2)	72 (23.2)	
Health care personnel	257 (58.9)	88 (13)	91 (20.9)	
Behvarz^b^	495 (63.1)	139 (17.7)	151 (19.2)	
Previous history of COVID-19				
Yes	790 (62.4)	256 (20.2)	221 (17.4)	0.286
No	1399 (62)	422 (18.7)	436 (19.3)	
History of hospitalization due to COVID-19				
Yes	97 (74.6)	20 (15.4)	13 (10)	0.007
No	2093 (61.6)	658 (19.4)	645 (19)	
Work experience, year				
≤ 10	1233 (61.4)	407 (20.3)	368 (18.3)	0.238
> 10	939 (63)	268 (18)	283 (19)	
Place of work				
Hospital	1145 (63.3)	346 (19.1)	317 (17.5)	
Primary health care center (urban)	515 (58.8)	173 (19.7)	188 (21.5)	0.211
Primary health care center (rural)	529 (62.8)	159 (18.9)	155 (18.4)	

^a^MD: Doctor of Medicine,

^b^Iranian rural health workers (Behvarz)

Table 3 displays multivariate logistic regression for socio-demographic predictators of intent to uptake the COVID-19 vaccine among health care workers. This finding indicates that 40–50 years old respondents were 1.56 times more likely to accept the vaccine (aOR: 1.56; 95% CI: 1.47–1.66). Similarly, participants with a history of COVID-19 infection were 0.85 times less likely to accept the vaccination (aOR: 0.85; 95% CI: 0.83–0.88). Respondents who were hospitalized due to COVID-19 were 2.18 times more likely to accept the vaccination infection (aOR: 2.18; 95% CI: 1.97–2.39) (Table 3).

**Table 3:** Multivariate logistic regression analysis for socio-demographic prediction of intent to uptake COVID-19 among Iranian HCWs (N = 3536)

Variables	“Intended to Uptake COVID-19 Vaccine”			
	OR [95% CI]	p-value	aOR [95% CI]	p-value
Sex				
Female	0.57 (0.55–0.59)	< 0.001	0.59 (0.57–0.61)	< 0.001
Male	Ref.		Ref.	Ref.
Marital status				
Married	1.09 (1.05–1.13)	< 0.001	0.99 (0.95–1.03)	0.56
Unmarried and widower	Ref.	Ref.	Ref.	Ref.
Age group, years old				
< 30	Ref.	Ref.	Ref.	Ref.
30–40	1.29 (1.24–1.34)	< 0.001	1.46 (1.39–1.53)	< 0.001
40–50	1.34 (1.29–1.4)	< 0.001	1.56 (1.47–1.66)	< 0.001
> 50	1.49 (1.39–1.59)	< 0.001	1.32 (1.21–1.43)	< 0.001
Education level				
High school diploma	Ref.	Ref.	Ref.	Ref.
Bachelors degree	0.86 (0.83–0.9)	< 0.001	0.87 (0.84–0.91)	< 0.001
Masters degree	0.7 (0.66–0.74)	< 0.001	0.68 (0.64–0.72)	< 0.001
MD	1.85 (1.75–1.96)	< 0.001	1.77 (1.67–1.88)	< 0.001
Work experience, year				
≤ 10	0.97 (0.94–0.99)	0.043	1.23 (1.17–1.29)	< 0.001
> 10	Ref.	Ref.	Ref.	Ref.
Previous history of COVID-19				
Yes	0.92 (0.87–0.95)	< 0.001	0.85 (0.83–0.88)	< 0.001
No	Ref.	Ref.	Ref.	Ref.
History of hospitalization due to COVID-19				
Yes	1.96 (1.78–2.15)	< 0.001	2.18 (1.97–2.39)	< 0.001
No	Ref.	Ref.	Ref.	Ref.

The distribution of preferred sources of information on COVID-19 vaccine by Iranian HCWs showed that the protocols and instructions issued by the Ministry of Health of Iran (60.4%), authentic medical information sources, scientific resources and sites (54.2%), Radio &Television(25.2%), and social networks (11.6%) were the most prioritized sources of information about COVID-19 for participants. Meanwhile, Iranian health managers and officials (59%), international managers and health officials (46.7%), and academic members (26.4%) were respectively influencer on their decision to get vaccinated.

## Discussion

Vaccination is vital for the prevention and elimination of the COVID-19 disease. The success of a vaccine relies on its efficacy and acceptance, primarily among health care workers (HCWs) who are at high risk of infection [17] with the essential role in the general population’s vaccination behaviors through their consultation [18]. Thus, we surveyed Iranian HCWs acceptance of the COVID-19 vaccine and the determinant factors.

Out of the 3536 participants, over 60% intended to uptake the COVID-19 vaccine, however both the hesitancy toward the COVID-19 vaccine and refusing to uptake the COVID-19 vaccine was reported about 19%. While at least in 3 other studies the number of people who did not want to get COVID-19 vaccinated was less than those who were hesitant to get vaccinated [18–20]. However in different studies, acceptance rates among HCWs ranged from almost 20–80% [18–24].

The COVID-19 vaccine acceptance in our participants (62%) was less than Gagneux-Brunon et al., study (76%), Whereas, in both our study and other studies, the acceptance of COVID-19 vaccine was less in nurses [21, 25]. Wang et al. and Fares et al. also reported that only 40.0% and 11.24% of nurses, respectively, intended to accept COVID-19 vaccination [17, 18].

Similar to several studies, in our study, older and male HCWs both showed more willingness to uptake the COVID-19 vaccine [24–26], while by increasing the years of education, acceptance of the COVID-19 vaccine increased [18, 21]. The highest chance of vaccine acceptance in our study and Qattan et al. [24] were in HCWs aged 40–50 years. However, Elhadi et al. and Fares et al. found that, the younger population were more ready to receive the vaccine [18, 22]. Our finding was not far-fetched, as HCWs are well informed that age is one of the influential risk factors for COVID-19 mortality [27–29]. Therefore, it was more probable that elder HCWs better accept the COVID-19 vaccine. We also found that in bivariate analysis, Doctors of Medicine were the most willing group to receive vaccination against COVID-19 (71.9%). Also, in multivariate logistic regression analysis, they were 1.77 times more likely to accept the vaccination (aOR: 1.77; 95% CI: 1.67–1.88). This finding was consistent with other studies, which found that, compared to other HCWs, Medical doctors were more likely to accept the COVID-19 vaccines if the vaccination was available [25, 30–33].

Our results suggested that both domestic and foreign vaccine products must be provided in order to increase healthcare workers' adherence. As only 28.7% of HCWs stated that they would prefer to receive the domestic vaccine. However Fu et al. demonstrated that 52.5% of Chinese HCWs believed that the domestic COVID-19 vaccine would be better than those produced abroad [9].

Our study revealed that only about 10% of HCWs said they did not trust any vaccine. However, trust in a vaccine is associated with vaccine up taking [34], and it is one of the critical attributes of vaccine hesitancy [34]. Quinn et al. reported that the trust was a strong and independent predictor of taking the flu vaccine [35]. On the other hand, many of those who refused or hesitated COVID-19 vaccination could accept vaccination in the future on the condition that they could trust [36]. In this regard, trust in authorities was a key factor [30]. Thus, the government and decision-makers had a critical role in creating and maintaining confidence, not only in the safety but also in the effectiveness of the vaccines during mass vaccination programs [37]. It seems that by offering the appropriate information about vaccines, decision-making would be facilitated, and the rate of vaccine acceptance would be increased as well.

On the other hand, a population survey of adults indicated that if the healthcare providers recommended vaccination, individuals would be more likely to receive a COVID-19 vaccine [38], which emphasized the role of health providers in the general acceptance of vaccines. So, health care providers and government officials should perform much effort to promote public trust and sincerity [37]. Shekhar et al. showed that HCWs who were vaccinated were more likely to recommend vaccines to others [20]. On the other hand, intent to be vaccinated was associated with trust in the health care system [39].

We also investigated the relationship among history of infection and hospitalization due to COVID-19 with vaccine uptake in HCWs. So that, in the multivariate logistic regression analysis, our results suggested that participants who had a history of COVID-19 infection were 0.85 times less likely to accept the vaccination (aOR: 0.85; 95% CI: 0.83–0.88). These results were similar to the study of Martin et al., in which they indicated that staff with a history of positive PCR were significantly less likely to be vaccinated. Maybe they believe that they have adequate immunological protection against COVID-19 infection. While, according to the evidence, the risk of infection may increase over time due to concerning waning humoral immunity [40]. So, given that HCWs are at the forefront of the fight against COVID-19, offering evidence-based information, could influence others to decide to receive the vaccine. Also, we found that respondents who were hospitalized due to COVID-19 were 2.18 times more likely to accept the vaccination (aOR: 2.18; 95% CI: 1.97–2.39). Probably, the perceived risk and severity of those HCWs who had an experience of hospitalization was higher than when COVID-19 infected them with low severity.

Our result demonstrated that the HCWs who had fewer work experiences were 1.23 times more likely to accept the vaccination; this finding was in agreement with the study by Papagiannis et al. They found that fewer work experiences enhanced the intention of HCWs to uptake the COVID-19 vaccine [41].

Studies indicated that trust in information sources was critical to vaccination acceptance [42]. The findings of this study showed that the most trustful sources of information about the COVID-19 vaccine in HCWs were the protocols and instructions of the Ministry of Health and authentic medical, scientific resources and sites. Regarding the sources of information in the Fares et al. [15] study, those who used the media as the source of information had the most negative COVID-19 vaccination perception. While, those who obtained information from published scientific articles had the most positive perception, although the difference between these groups was insignificant.

We found that the recommendations of Iranian (domestic) health managers and officials, international managers and health officials, and academic members were influential in HCWs decision to receive the vaccine. Meanwhile, Rozek et al. indicated that individuals who had confidence in international organizations such as WHO was more likely to accept vaccine than those who reported no confidence in the WHO. Trust in health scientists and local and national health ministries were also predictive of reduced vaccine hesitancy [43]. Research on the vaccination of HCWs against pandemic H1N1 showed that they were influenced by positive external cues to action, such as physicians and supervisors [44].

### Strengths and limitations

There are several strengths in the present study. First, participants were recruited and surveyed in face-to-face interviews. A multi-stage cluster sampling method by random sampling method was used according to healthcare worker statistics for gender and occupation from each medical sciences university, which led to less bias than online surveys. Second, the sample size was large, and data from a multicenter could affect the generalizability. Third, our study had many findings, which would help policymakers in the national COVID-19 vaccination program. Fourth, in the present study, we simultaneously focused on both hospital and primary health care workers (urban and rural).

There were some limitations in the present study. It should be considered that all surveys were snapshots taken at a point in time. So, they give us an idea of how participants are doing at the time of the survey. Thus, participants' perceptions of receiving the vaccine may change with the COVID-19 pandemic trend. However, this information can guide health policy makers and stakeholders in planning health interventions. Furthermore, we sought to evaluate the intention of HCWs toward accepting a vaccine while the vaccination program was not completely started yet. Hence, as more information becomes attainable on the effectiveness and safety of COVID-19 vaccines and access to various vaccines, participants might change their amind regarding vaccination.

## Conclusion

In total, 62% of our participants were willing to uptake vaccination against COVID-19 as soon as it was available. This study showed a moderate acceptance of COVID-19 vaccination in the HCWs in the Islamic Republic of Iran. Several factors influenced HCWs’ vaccine acceptance.

It needs to pay more attention to and implementation of educational programs for increasing awareness and reassurance of HCWs and affecting their attitudes toward COVID-19 vaccination. Health promotion strategies need to address the infodemy and vast misinformation about COVID-19 vaccines. Moreover, clear communication about vaccine safety and effectiveness will increase HCWs trust in COVID-19 vaccination programs. It is suggested that by targeting HCWs, the organizational culture of vaccination in the workplace creates and is strengthened through strong messages from managers, health officials, and academic members in Iran.

## Supplementary Information

**Additional file 1. Appendix1: **The flow chart of recruiting HCWs in Iranian Medical sciencesUniversities.

## Data Availability

The data that support the findings of this study are available from [Center for Communicable Diseases Control, Ministry of Health and Medical Education of Iran), but restrictions apply to the availability of these data, which were used under license for the current study, and so are not publicly available. Data are however available from the authors upon reasonable request and with permission of [Center for Communicable Diseases Control, Ministry of Health and Medical Education of Iran].
