# Supplementary material for: Determinants of COVID-19 vaccine acceptance in healthcare workers in Iran: National Survey
Source: BMC Infect Dis. 2022 Aug 22;22:703. doi: 10.1186/s12879-022-07675-x (PMC9395894; doi:10.1186/s12879-022-07675-x)
Supplement: Supplementary file 1 — Additional file 1. Appendix1: The flow chart of recruiting HCWs in Iranian Medical sciencesUniversities. [file 12879_2022_7675_MOESM1_ESM.docx]

Appendex1: The flow chart of recruiting HCWs in Iranian Medical sciences Universities

The Universities Of Medical Sciences in the Islamic Republic of Iran

(N=60)

Random selection of the Universities Of Medical Sciences (N=9)

UMS1

UMS2

UMS3

UMS4

UMS5

UMS6

UMS7

UMS9

UMS8

Random selection of two cities covered by each University of Medical Sciences

Selecting 10 (PHC center**^*^**):

5 PHC centers from City1 (rural and urban)

5 PHC centers from City2 (rural and urban)

Selecting 2 Hospitals:

The COVID-19 centeral hospital from city 1 and a general hospital from city 2

Respondents were selected by random sampling method according to healthcare worker statistics for gender and occupation from each university of medical sciences (n= 400: 200 from Hospital, 200 from PHC centers)

**^*^**According to the structure of PHC system in Iran, each rural (sometimes a group of rural) has a health-house, where community health woekres named Behvarz are working there. Each two behvarz are cover health services of 1200 inhabitants. These health-houses provide the first level preventive health services to the inhabitants. Every 3-5 health house are supervised by a rural health center which includes a GP and a health team that provides health care for more complex health problems. In urban PHCs health personnel’s provide similar services as health-houses and rural PHCs. This system is managed by district health centers, under the supervision of Medical Sciences Universities. In each province, there is at least one Medical Sciences University(47).
